# Advancing Interpretable Regression Analysis for Binary Data: A Novel Distributed Algorithm Approach

**DOI:** 10.1002/sim.10250

**Published:** 2024-11-03

**Authors:** Jiayi Tong, Lu Li, Jenna Marie Reps, Vitaly Lorman, Naimin Jing, Mackenzie Edmondson, Xiwei Lou, Ravi Jhaveri, Kelly J. Kelleher, Nathan M. Pajor, Christopher B. Forrest, Jiang Bian, Haitao Chu, Yong Chen

**Affiliations:** ^1^ Center for Health AI and Synthesis of Evidence (CHASE), Department of Biostatistics, Epidemiology and Informatics, Perelman School of Medicine University of Pennsylvania Philadelphia Pennsylvania USA; ^2^ Department of Biostatistics Johns Hopkins Bloomberg School of Public Health Baltimore Maryland USA; ^3^ The Graduate Group in Applied Mathematics and Computational Science, School of Arts and Sciences University of Pennsylvania Philadelphia Pennsylvania USA; ^4^ Janssen Research and Development Titusville New Jersey USA; ^5^ Observational Health Data Sciences and Informatics (OHDSI) New York New York USA; ^6^ Department of Medical Informatics Erasmus University Medical Center Rotterdam The Netherlands; ^7^ Applied Clinical Research Center, Children's Hospital of Philadelphia Philadelphia Pennsylvania USA; ^8^ Biostatistics and Research Decision Sciences, Merck & Co., Inc Rahway New Jersey USA; ^9^ Health Outcomes & Biomedical informatics, College of Medicine University of Florida Gainesville Florida USA; ^10^ Division of Infectious Diseases Ann & Robert H. Lurie Children's Hospital of Chicago Chicago Illinois USA; ^11^ Center for Child Health Equity and Outcomes Research The Abigail Wexner Research Institute at Nationwide Children's Hospital Columbus Ohio USA; ^12^ Divisions of Pulmonary Medicine | Biomedical Informatics | James M. Anderson Center for Health Systems Excellence Cincinnati Children's Hospital Medical Center and University of Cincinnati College of Medicine Cincinnati Ohio USA; ^13^ Statistical Research and Data Science Center, Pfizer Inc. New York New York USA; ^14^ Department of Biostatistics, Epidemiology and Informatics, Perelman School of Medicine University of Pennsylvania Philadelphia Pennsylvania USA; ^15^ Penn Institute for Biomedical Informatics (IBI) Philadelphia Pennsylvania USA; ^16^ Leonard Davis Institute of Health Economics Philadelphia Pennsylvania USA; ^17^ Penn Medicine Center for Evidence‐based Practice (CEP), Philadelphia Pennsylvania USA

**Keywords:** binary data, distributed algorithm, modified Poisson regression, relative risk

## Abstract

Sparse data bias, where there is a lack of sufficient cases, is a common problem in data analysis, particularly when studying rare binary outcomes. Although a two‐step meta‐analysis approach may be used to lessen the bias by combining the summary statistics to increase the number of cases from multiple studies, this method does not completely eliminate bias in effect estimation. In this paper, we propose a one‐shot distributed algorithm for estimating relative risk using a modified Poisson regression for binary data, named ODAP‐B. We evaluate the performance of our method through both simulation studies and real‐world case analyses of postacute sequelae of SARS‐CoV‐2 infection in children using data from 184 501 children across eight national academic medical centers. Compared with the meta‐analysis method, our method provides closer estimates of the relative risk for all outcomes considered including syndromic and systemic outcomes. Our method is communication‐efficient and privacy‐preserving, requiring only aggregated data to obtain relatively unbiased effect estimates compared with two‐step meta‐analysis methods. Overall, ODAP‐B is an effective distributed learning algorithm for Poisson regression to study rare binary outcomes. The method provides inference on adjusted relative risk with a robust variance estimator.

## 1 Introduction

Sparse data are a major challenge in data analysis, particularly when analyzing rare binary outcomes [1, 2]. The lack of sufficient cases in the data can lead to biased estimates of treatment effects in observational studies and clinical trials [3, 4, 5]. To tackle the bias due to sparse data, the traditional two‐step meta‐analysis is commonly used to combine information from multiple studies (i.e., analyze each individual dataset first, then combine a common effect, a fixed effect, or a random effects model [6]), often in settings where some studies have relatively small sample sizes. Meta‐analysis provides a summary estimate with greater statistical precision than the estimate from any individual study. However, it has been shown that the sparse data bias in effect estimation is not completely eliminated by using meta‐analysis [3]. While Individual Participant Data (IPD) meta‐analysis can increase the number of cases by pooling small samples together, it is often infeasible to pool these data across studies due to logistical, regulatory, privacy, and other concerns. To address these challenges, distributed algorithms for sparse binary outcome data (hereinafter referred to as binary data) are desirable, particularly distributed learning algorithm which has comparable communication costs as the traditional meta‐analysis [7].

The logistic regression model is a popular choice for analyzing binary data. It produces an odds ratio (OR) to measure the effect of an intervention or the strength of an association between two groups. Another metric commonly used in biomedical studies for binary outcomes is the relative risk (RR). The choice between RR and OR has been debated in the literature, with RR being preferred in most prospective studies due to its collapsibility and better interpretation, particularly when the outcome is not rare [7, 8, 9, 10, 11, 12, 13, 14, 15].

There are two approaches to estimating RR: conversion from estimated OR and direct estimation. Studies have shown that direct estimation is preferred as it produces more reliable confidence limits and consistent estimates [16]. Poisson regression is typically recommended to estimate the adjusted RR directly for binary data, as the Poisson distribution can approximate the binomial distribution when the sample size is large and the probability is small [17, 18]. A modified Poisson regression with a sandwich error term, proposed by Zou, allows for direct estimation of the adjusted RR with robust variance estimation, even when the Poisson model is misspecified for the binary outcome [18, 19, 20].

In this manuscript, we present a one‐shot distributed algorithm for the modified Poisson regression model with a focus on the analysis of rare binary data, which poses complications due to the natural bound of parameters for common outcomes [12]. We define “one‐shot” as a process where the lead site is involved in a single round of communication—beginning with receiving initial estimates, followed by broadcasting the overall initial value and concluding with receiving gradients from local sites. This constitutes a complete communication cycle within the implementation procedure. Our algorithm requires only aggregated data and allows for the estimation of RR using Poisson regression without the need for individual‐level data. The proposed method provides consistent estimates of intervention effects and robust variance estimation of the estimated RR through sandwich estimation. We evaluated the performance of our method through extensive simulation studies and compared the estimates with those obtained using two‐step meta‐analysis and pooled methods, where data from multiple sites are pooled together. Additionally, we conducted two use cases with real‐world data on postacute sequelae of SARS‐CoV‐2 infection (PASC). The method is available in the R package ‘pda’, as well as in STATA and SAS code in the web appendix for dissemination.

## 2 Methods

**FIGURE 1:**
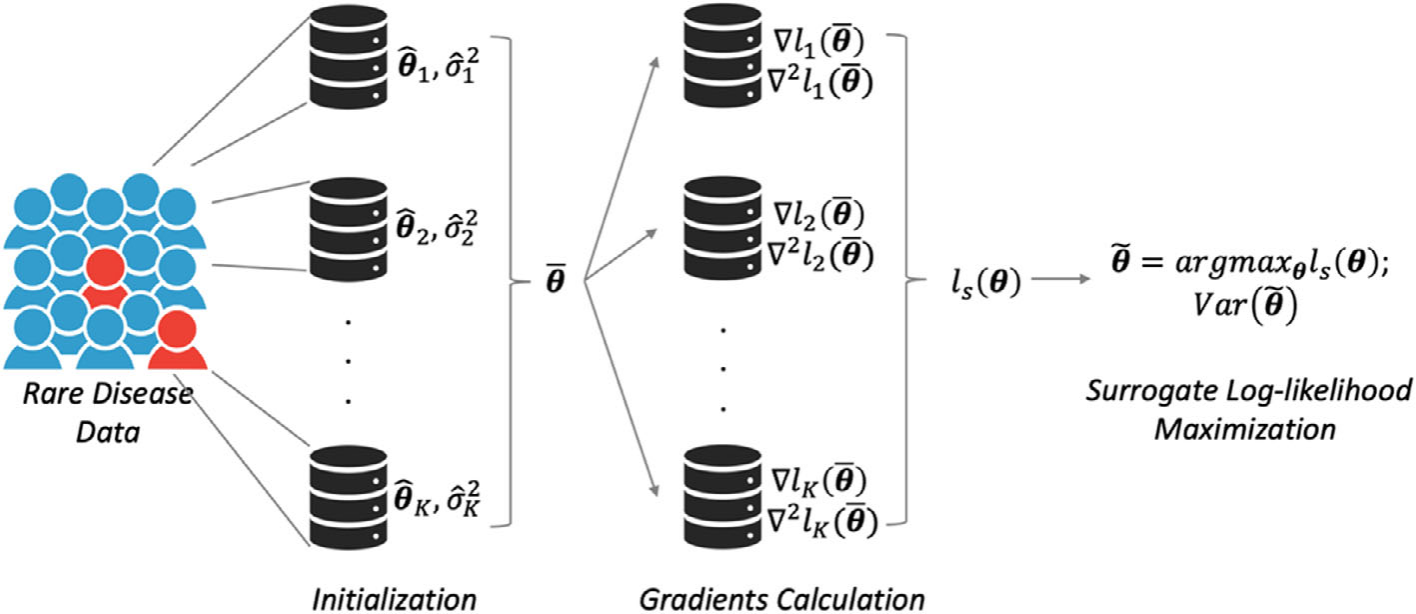
Schematic illustration of the proposed ODAP‐B method. Each site calculates the initial estimate ${\hat{\theta }}_{k}$ and variance ${\hat{\sigma }}_{k}^{2}$ in the initialization step. Then, the meta‐estimate $\bar{\theta }$ is obtained and transferred to all sites for the gradient calculation. Each site calculates the aggregated data (i.e., first and second gradients) with the initial value and local data, and then transfers the gradients back to the lead site or local site for the construction of the surrogate likelihood function.

We propose a one‐shot distributed modified Poisson regression approach, referred to as ODAP‐B, for binary data. The workflow of the proposed algorithm is illustrated in Figure 1. Assume that there are *K* sites in total that contribute data to the federated learning study. For simplicity, we assume Site 1 serves as the lead site, where the patient‐level data at Site 1 is accessible. For Site 2 to Site *K*, the patient‐level data are not allowed to be shared, but aggregated data.

Let $Y_{\mathrm{kj}}$ denote the binary outcome variable of the j‐th patient from the site $k\;\left(k=1,\ldots ,K\right),j=\left(1,2,\ldots ,n_{k}\right)$, where $n_{k}$ is the sample size of the k‐th site. $Y_{\mathrm{kj}}=1$ if the outcome occurs and $Y_{\mathrm{kj}}=0$ otherwise. Let $X_{\mathrm{kj}}$ be the binary exposure of interest (e.g., medications, treatments, or drugs) and $Z_{\mathrm{kj}}$ be the vector of confounding variables (e.g., age, gender, race, etc.) of the j‐th patient from the k‐th site. To directly estimate the RR of the binary outcome comparing *X* = 1 versus *X* = 0, the logarithm link function is used:

$$\log\left[P\left(X_{\mathrm{kj}},Z_{\mathrm{kj}}\right)\right]=\alpha +\beta X_{\mathrm{kj}}+\gamma Z_{\mathrm{kj}}$$

where exp(α) is the risk of outcome for the case where the exposure and variables are all zero, exp(β) is the RR of the exposure of interest, and exp(γ) is the vector of relative risks of the confounding variables. If a Poisson distribution is assumed for the outcome (i.e., $Y_{\mathrm{kj}}$), the pooled log‐likelihood function for all *K* sites is given by:

(1) $$l\left(\alpha ,\beta ,\gamma \right)=\frac{1}{N}\sum_{k=1}^{K}\sum_{j=1}^{n_{k}}\left[Y_{\mathrm{kj}}\left(\alpha +\beta X_{\mathrm{kj}}+\gamma Z_{\mathrm{kj}}\right)-\exp\;\left(\alpha +\beta X_{\mathrm{kj}}+\gamma Z_{\mathrm{kj}}\right)\right]$$

where $N=\sum_{k=1}^{K}n_{k}$ is the total sample size. By maximizing the pooled log‐likelihood function, the pooled estimates of the parameters can be obtained.

However, individual patient‐level data often cannot be shared across studies done at different institutions due to patient privacy, regularity, and confidentiality concerns. As a result, we cannot directly obtain the pooled log‐likelihood function for all *K* sites. Within each site, we can construct the local log‐likelihood function only. For the *k*‐th site, the local log‐likelihood function is given by:

(2) $$l_{k}\left(\alpha ,\beta ,\gamma \right)=\frac{1}{n_{k}}\sum_{j=1}^{n_{k}}\left[Y_{\mathrm{kj}}\left(\alpha +\beta X_{\mathrm{kj}}+\gamma Z_{\mathrm{kj}}\right)-\exp\;\left(\alpha +\beta X_{\mathrm{kj}}+\gamma Z_{\mathrm{kj}}\right)\;\right]$$

Using methods developed by Jordan et al. [21] and adapted to the clinical setting by Duan et al. [22, 23], we implemented a surrogate likelihood approach, by constructing the surrogate log‐likelihood function at the lead site.

Since patient‐level data from the collaborating sites cannot be accessed, the surrogate likelihood function approximates the pooled likelihood function by using summary‐level statistics from collaborating sites. The key technique used in the construction of the surrogate likelihood function is Taylor expansion. In particular, by applying the Taylor expansion to both the pooled likelihood function and the local likelihood function (i.e., the likelihood function for Site 1) around the same initial value $\bar{\theta }$, the higher‐order terms of the local likelihood can be used to approximate the higher‐order terms of the pooled likelihood This results in the following surrogate likelihood function after dropping the constant terms. Let θ = (α,β,γ), the surrogate log‐likelihood is defined as:

(3) $$\begin{array}{cc} l_{s}\left(\theta \right) & =l_{1}\left(\theta \right)+{\left\{\nabla l_{K}\left(\bar{\theta }\right)-\nabla l_{1}\left(\bar{\theta }\right)\right\}}^{T}\\ & \;\theta +\frac{1}{2}\;{\left(\theta -\bar{\theta }\right)}^{T}\left\{\nabla^{2}l_{K}\left(\bar{\theta }\right)-\nabla^{2}l_{1}\left(\bar{\theta }\right)\right\}\left(\theta -\bar{\theta }\right) \end{array}$$

where $\;l_{1}$ is the log‐likelihood of the lead site (i.e., Site 1), $\nabla l_{K}\left(\bar{\theta }\right)$ and $\nabla^{2}l_{K}\left(\bar{\theta }\right)$ are the averages of all *K* sites' first gradients and second gradients of log‐likelihood functions, respectively, calculated as follows:

$$\nabla l_{K}\left(\bar{\theta }\right)=\frac{1}{K}\sum_{k=1}^{K}\nabla l_{k}\left(\bar{\theta }\right)$$

$$\nabla^{2}l_{K}\left(\bar{\theta }\right)=\frac{1}{K}\sum_{k=1}^{K}\nabla^{2}l_{k}\left(\bar{\theta }\right)$$

$\bar{\theta }$ is the initial value, which can be obtained by using the local estimate or a meta‐estimate of θ. The first gradient of the k‐th site is calculated as:

$$\nabla l_{k}\left(\bar{\theta }\right)=\frac{1}{n_{k}}\sum_{j=1}^{n_{k}}X_{\mathrm{kj}}\left(Y_{\mathrm{kj}}-\exp\;\left\{X_{\mathrm{kj}}^{T}\bar{\theta }\right\}\;\right),$$

and the second gradient is calculated as:

$$\nabla^{2}l_{k}\left(\bar{\theta }\right)=-\frac{1}{n_{k}}\sum_{i=1}^{n_{k}}\exp\;\left(X_{\mathrm{kj}}^{T}\bar{\theta }\right)\;X_{\mathrm{kj}}X_{\mathrm{kj}}^{T},$$

where $X_{\mathrm{kj}}=\left(1,X_{\mathrm{kj}},Z_{\mathrm{kj}}\right)$. The proposed ODAP‐B estimator is given as:

(4) $$\tilde{\theta }={\text{argmax}}_{\theta }l_{s}\left(\theta \right)$$

For the variance estimate of $\tilde{\theta }$, the sandwich estimator is used due to the misspecification of the data distribution which should be binomial. The consistent estimator of the variance of $\tilde{\theta }$ is given by:

$$\mathrm{Var}\left(\tilde{\theta }\right)=\frac{n_{1}}{N}{\left(\nabla^{2}l_{1}\left(\tilde{\theta }\right)\right)}^{-1}\nabla l_{1}\left(\tilde{\theta }\right){\left(\nabla l_{1}\left(\tilde{\theta }\right)\right)}^{T}{\left(\nabla^{2}l_{1}\left(\tilde{\theta }\right)\right)}^{-1}.$$

This procedure *preserves patient‐level privacy* in the data integration process by only transferring aggregated data across sites. It is important to note that any of the sites participating in the study can serve as the lead site to construct the above surrogate likelihood function and obtain the ODAP‐B estimator. In practice, we can have each site serve as the lead site and perform the proposed distributed learning algorithm. The estimators from all sites can then be combined using a meta‐analysis method.

Unlike the traditional meta‐analysis method, where synthesized estimates can be biased if local estimates are biased due to an ill‐behaved likelihood function in the presence of data sparsity, the proposed method is able to handle rare events well as it is a likelihood‐based approach. By incorporating the gradients of the likelihood into the surrogate likelihood, the shape of the local likelihood is accurately described and considered, leading to more robust estimates even when data sparsity is present. The superior performance of handling rare events by incorporating the surrogate likelihood function in the distributed learning method has been demonstrated in several existing studies [24, 25, 26, 27].

### 2.1 Implementation of ODAP‐B Algorithm

The implementation steps are summarized in Table 1. To implement the ODAP‐B algorithm in a real‐world setting, the first step (i.e., initialization step) is to fit the modified Poisson regression model within each local site on the local patient‐level data to obtain the initial estimates and standard errors of the regression coefficients. These values are then transferred to the lead site (i.e., Site 1), where Site 1 synthesizes the local initial estimates to obtain a meta‐analytical estimate. This estimate is used as the overall initial value for the following steps. This meta‐analysis step is optional, meaning that a local initial value can be used as the overall initial value. However, to ensure the reliability of the overall initial value, the meta‐analytical estimate is more robust as suggested in Duan et al. [24], especially in the presence of relatively small sample sizes at local sites.

**TABLE 1:** The implementation steps for the proposed ODAP‐B algorithm.

Proposed algorithm: ODAP‐B
1. **Step 1 (Initialization)** For Site 1 to Site K: **do**: Obtain local initial value: $${\bar{\beta }}_{k}=\arg\max_{\beta }\;l_{k}\left(\beta \right)$$ where $l_{k}\left(\beta \right)$ is the log‐likelihood of the modified Poisson regression model of the k‐th site; Transfer local initial value to the lead site (i.e.,Site 1). **end**
2. **Step 2 (Meta‐analysis for overall initial value)** At Site 1 (i.e., Lead site): **do:** Synthesize the local initial value through meta‐analysis to obtain the overall initial value; Broadcast the overall initial value to all sites. **end**
3. **Step 3 (Generation & Transfer of aggregated data)** For Site 1 to Site K: **do**: Compute the first gradient $\nabla l_{k}\left(\bar{\theta }\right)$ and second gradient $\nabla^{2}l_{k}\left(\bar{\theta }\right)$; Transfer the aggregated data (i.e., gradients) to the lead site. **end**
4. **Step 4 (Construction of surrogate likelihood and calculation of final estimator)** At Site 1 (i.e., Lead site): **do:** Construct $l_{s}\left(\theta \right)$, obtain $\tilde{\theta }={\text{argmax}}_{\theta }l_{s}\left(\theta \right)$ and $\mathrm{Var}\left(\tilde{\theta }\right)$ **end**

The lead site then broadcasts the overall initial value to all collaborating sites. These sites produce the first and second gradients of their local log‐likelihood with the overall initial value, constructed using their patient‐level data. These gradients are transferred back to the lead site (i.e., Site 1), where the proposed surrogate likelihood function is constructed. The ODAP‐B estimator is then obtained by maximizing this function, as presented in Equation (4).

It is crucial to clarify that the implementation procedure engages the local sites in two specific instances: (1) transferring initial local values to the lead site, and (2) transferring gradients back to the lead site. Notably, the first transfer step is optional. If users opt to use the initial value from a single local site as the overall initial value, this leads to only one instance of transferring aggregated data from the local sites. Our proposed method is defined as a “one‐shot” algorithm, given that it only requires a closed communication cycle beginning with receiving initial estimates, followed by broadcasting the overall initial value and concluding with receiving gradients from local sites.

## 3 Simulation Study

To evaluate the proposed method, we conducted simulation studies and compared our proposed ODAP‐B method with the two‐step meta‐analysis method and the pooled analysis. The two‐step meta‐analysis involves first analyzing the patient‐level data separately for each site to obtain relevant local estimates with standard errors. In the second step, these local estimates are combined to produce summary results. Pooled analysis refers to the ideal setting where all patient‐level data from the sites are available. In this case, the log‐likelihood function (1) can be constructed to obtain the pooled estimates. We compared the estimation bias of the regression coefficients by our method and the meta‐analysis method relative to the pooled analysis. The metric for this comparison is the relative bias to the pooled analysis.

**FIGURE 2:**
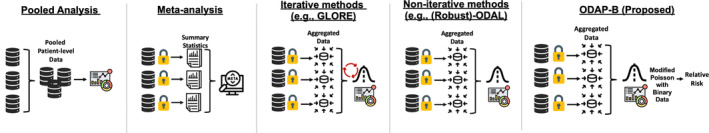
Workflows of existing methods and the proposed ODAP‐B method. The pooled analysis assumes all patient‐level data can be combined for analysis. Meta‐analysis fits models separately at each site and combines local estimates. Iterative methods share aggregated data across sites until convergence, while noniterative methods fit the model within a limited number of communication rounds. The proposed ODAP‐B method, which is a noniterative method, fits modified Poisson regression in a distributed manner, requiring only aggregated data.

Table 2 and Figure 2 show the comparisons between the pooled analysis, the meta‐analysis method, existing distributed algorithms for the logistic regression model, and the proposed method from various aspects. Our proposed method, which is based on the surrogate likelihood approach, is able to retain high accuracy in estimating model parameters and protect patient privacy while being communication efficient when estimating the association between exposures and outcome of interest in the case of relatively rare binary outcomes.

**TABLE 2:** Summary of existing methods comparison.

	Accuracy	Privacy protection?	Communication efficient?	Capable of handling rare event?	Collapsibility of effect
Pooled Analysis^a^	High	NO (patient‐level data are shared)	NO (large amount of patient‐level data are shared)	YES	YES
Meta Analysis^b^	Varying (Not accurate for rare diseases)	YES	YES	NO	YES
GLORE [44]	High	YES	NO (iterative algorithms)	YES	NO (odds ratio is obtained)
(Robust)‐ODAL, dCLR^c^ [22, 45, 46, 47]	High	YES	YES	YES	NO (odds ratio is obtained)
ODAP‐B	**High**	**YES**	**YES**	**YES**	**YES**

*Note*: Comparisons among pooled analysis, meta‐analysis, distributed algorithms for a logistic regression model, and the proposed method. Accuracy is evaluated through mean squared error (MSE) and bias to the true value: The smaller the MSE or bias is, the better the accuracy is. Privacy is evaluated based on whether the method is an aggregated data‐based approach without sharing patient‐level information. The evaluation of communication is through the number of rounds of transferring aggregated data across sites and the number of digits to be communicated within each round.

^a^ Pooled analysis: Fitting the modified Poisson regression on the pooled data.

^b^ Meta‐analysis: Modified Poisson regression is fitted within each site and then meta‐analyses the summary statistics to obtain pooled RR.

^c^ ODAL: One‐shot distributed algorithm for logistic regression; dCLR: Distributed algorithm for the conditional logistic regression model [22, 45, 46, 47].

In the simulation study, we set the total number of sites to be 5 or 50, which is consistent with the size of many clinical research networks in the United States. The sample size of each site was set to 500, leading to a total sample size across all sites to be 2500 or 25 000. We consider the setting where a binary outcome is associated with four variables, including three binary predictors (e.g., medication, sex, chronic condition) and one continuous predictor (e.g., age). The exposure of interest (e.g., medication) was generated from a Bernoulli distribution with a probability of 0.3. The second and third binary predictors were generated from Bernoulli distributions with probabilities 0.6 and 0.5, respectively, which were motivated by “sex” in the pediatric PASC data. The continuous predictor was sampled from the “age” variable in the pediatric PASC data to be elaborated in the Data Application Section. With these predictors, we have the following model to fit:

$$\log\left(P\left(Y=1|X,Z\right)\right)=\alpha +\beta_{1}X_{1}+\gamma_{1}Z_{1}+\gamma_{2}Z_{2}+\gamma_{3}Z_{3},$$

where the true values of the parameters are set as α=−2or−5 to mimic a relatively common disease and rare disease with a prevalence of 12% and 0.7%, respectively. $\beta_{1}=-0.25\;\text{or}-1$, and $\gamma_{1}=\gamma_{2}=\gamma_{3}=-0.1$. The simulation was conducted with 1000 replications.

**FIGURE 3:**
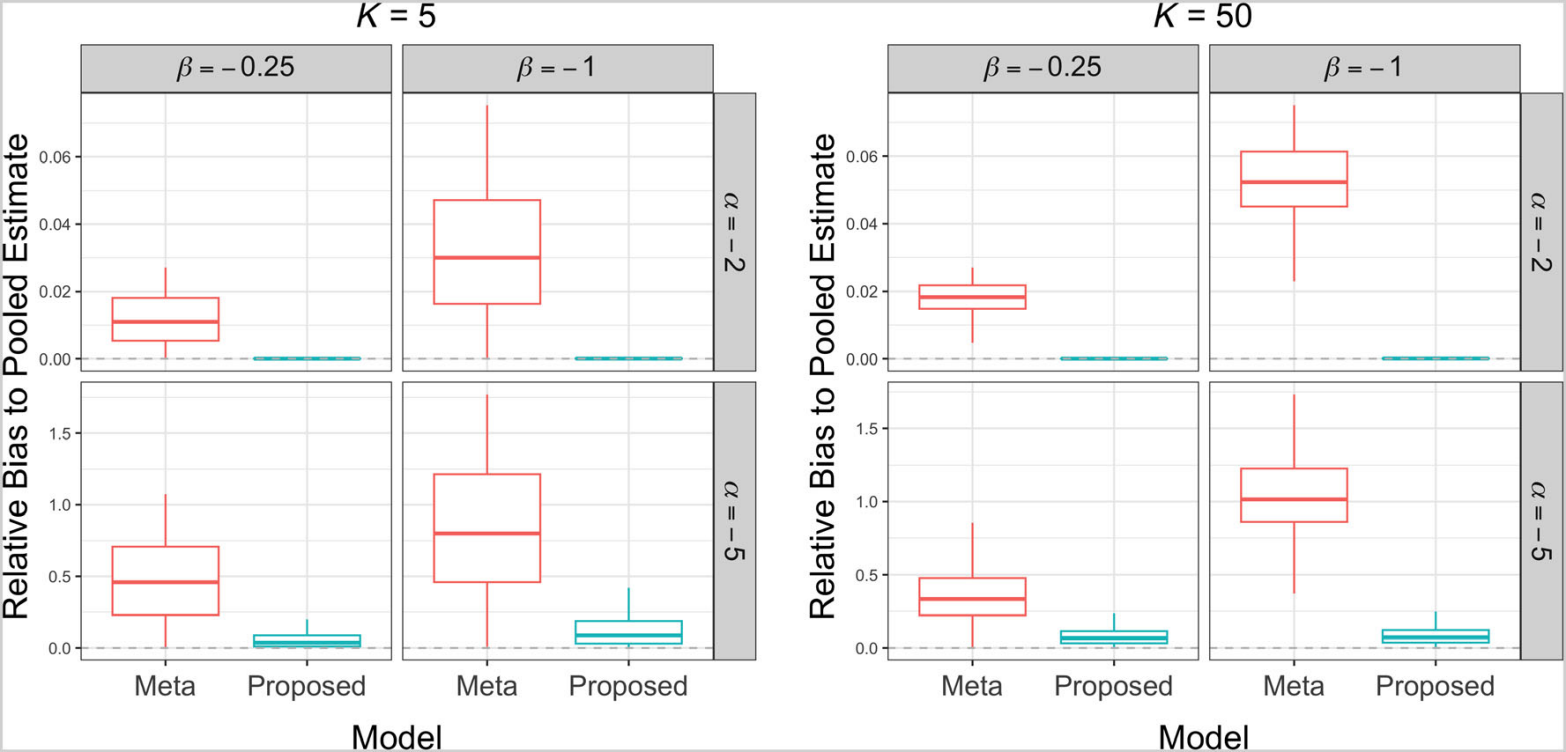
Comparison between the meta‐analysis method (left box, red) and the proposed ODAP‐B method (right box, cyan) in terms of relative bias of $\beta_{1}$ to the gold standard pooled analysis when the total number of sites is 5 (left panel) and 50 (right panel).

Figure 3 shows the simulation results. The left panel presents the case where the total number of sites is 5 (i.e., *K* = 5) and the right panel presents the case where the total number of sites is 50 (i.e., *K* = 50). Within each panel, the two vertical box plots on the left are the results when the intercept (i.e., α) is −2, which represents a relatively common disease; while the two vertical box plots on the right in both figures are the results for the intercept equal to −5, which mimics a relatively rare disease scenario. In each figure, we compared the relative bias of the proposed estimate (cyan, right) and the meta‐estimate (red, left) of the exposure of interest's effect ($\beta_{1}$) with respect to the pooled one.

As shown in Figure 3, when the outcome prevalence is relatively common, that is, α=−2, the meta‐analysis method and the proposed ODAP‐B method perform similarly, and both are close to the pooled method. As the event rate decreases, the meta‐analysis method (red, left) is observed to have a larger relative bias compared with the proposed method (cyan, right). Wider boxes of the meta‐analysis method compared with those of the proposed method also present the lack of efficiency of the meta‐analysis method in estimating the effect of exposure of interest compared with the proposed method.

We further examined the preservation of type‐I error of the proposed method and found that for most of the settings, the proposed method maintains an acceptable level of type‐I error and yields values closer to those of the pooled analysis compared to the meta‐analysis method. The details description and results are available in Section S3.

## 4 Validation and Evaluation

To demonstrate the performance and validate the applicability of the proposed method, we conducted two use cases using real‐world clinical data:

- Use case 1: **centralized** data from PEDSnet
  ○ Participating sites: nine pediatric hospitals from the PEDSnet (including Cincinnati Children's Hospital Medical Center, Seattle Children's Hospital, Stanford Children's Health, Children's Hospital Colorado, Lurie Children's Hospital of Chicago, Nemours Children's Health System, Nationwide Children's Hospital, Children's Hospital of Philadelphia) contributed centralized data, hosted at the Children's Hospital of Philadelphia (CHOP)
- Use case 2: **decentralized** data from PEDSnet, Janssen, and OneFlorida+.
  ○ Participating sites: Janssen Pharmaceutical Companies of Johnson & Johnson and the OneFlorida+ Clinical Research Network (i.e., another network like PEDSnet with 14 different partners across Florida, Georgia, and Alabama)

We aimed to investigate the relationship between COVID‐19 viral (SARS‐CoV‐2 polymerase chain reaction [PCR] or antigen) test positivity and the symptoms and conditions associated with PASC in children [28]. In the Researching COVID to Enhance Recovery (RECOVER) program, PASC is defined as ongoing, relapsing, or new symptoms, or other health effects occurring after the acute phase of SARS‐CoV‐2 infection (i.e., present four or more weeks after the acute infection). There is a small but growing literature investigating pediatric PASC as well as an expanding literature on PASC in adults [29, 30, 31, 32]. Our understanding of the clinical features and long‐term impact of COVID‐19 in children is still limited.

In use case 1, where the patient‐level data from all nine sites are centralized at a central server, we compared the relative risks estimated using the meta‐analysis method, the proposed ODAP‐B method, and the gold standard method (modified Poisson regression using the pooled data). The proposed ODAP‐B method outperforms the meta‐analysis method by providing relative risk estimates that are closer to those from the gold standard method, especially given the fact that the meta‐analysis method has a poor performance in analyzing rare events as shown in the simulation studies.

In use case 2, no gold standard is available because the patient‐level data are stored locally within each site and only aggregated data are allowed to be shared, leading to that applying the pooled analysis is not feasible. Such a setting is referred to as a decentralized setting. Under such a setting, we applied our method to the data and estimated the effect size of the exposure. As there is no gold standard value to compare with, we compared the estimated relative risks with those from use case 1 to examine the consistency of findings obtained from two different databases.

### 4.1 Use Case 1: Application With a Centralized Dataset of 184 501 Children Across eight National Academic Medical Centers Within PEDSnet

In this use case, we used electronic health record (EHR) data from PEDSnet [33], a national clinical research network of large pediatric medical centers, to investigate the association between viral positivity and various outcomes related to PASC in children.

The data were retrieved from all inpatient, outpatient, and emergency department healthcare encounters associated with patients who underwent SARS‐CoV‐2 PCR or antigen testing provided in any of these settings. The data were extracted from the PEDSnet COVID‐19 Database‐Version 2022‐01‐16.

Cohort entrance was defined as the day of the first SARS‐CoV‐2 PCR or antigen test. The study cohort includes individuals under 21 years of age at the cohort entrance. The study period was from March 1, 2020, to October 31, 2021, from which the data were extracted.

The exposed cohort consisted of patients who were under 21 years of age at the time of the health encounter and who had a positive SARS‐CoV‐2 viral test between March 1, 2020 and October 31, 2021. The unexposed cohort was defined as patients with a negative SARS‐CoV‐2 viral test and no positive tests during the study period. The cohort was further restricted to patients who had at least one encounter, including telehealth visits, radiology or lab encounters, or administrative/telephone encounters, at a PEDSnet site between 3 years and 7 days prior to cohort entry. Based on these criteria, a study sample was selected for analysis with a sample size of 184 501.

**FIGURE 4:**
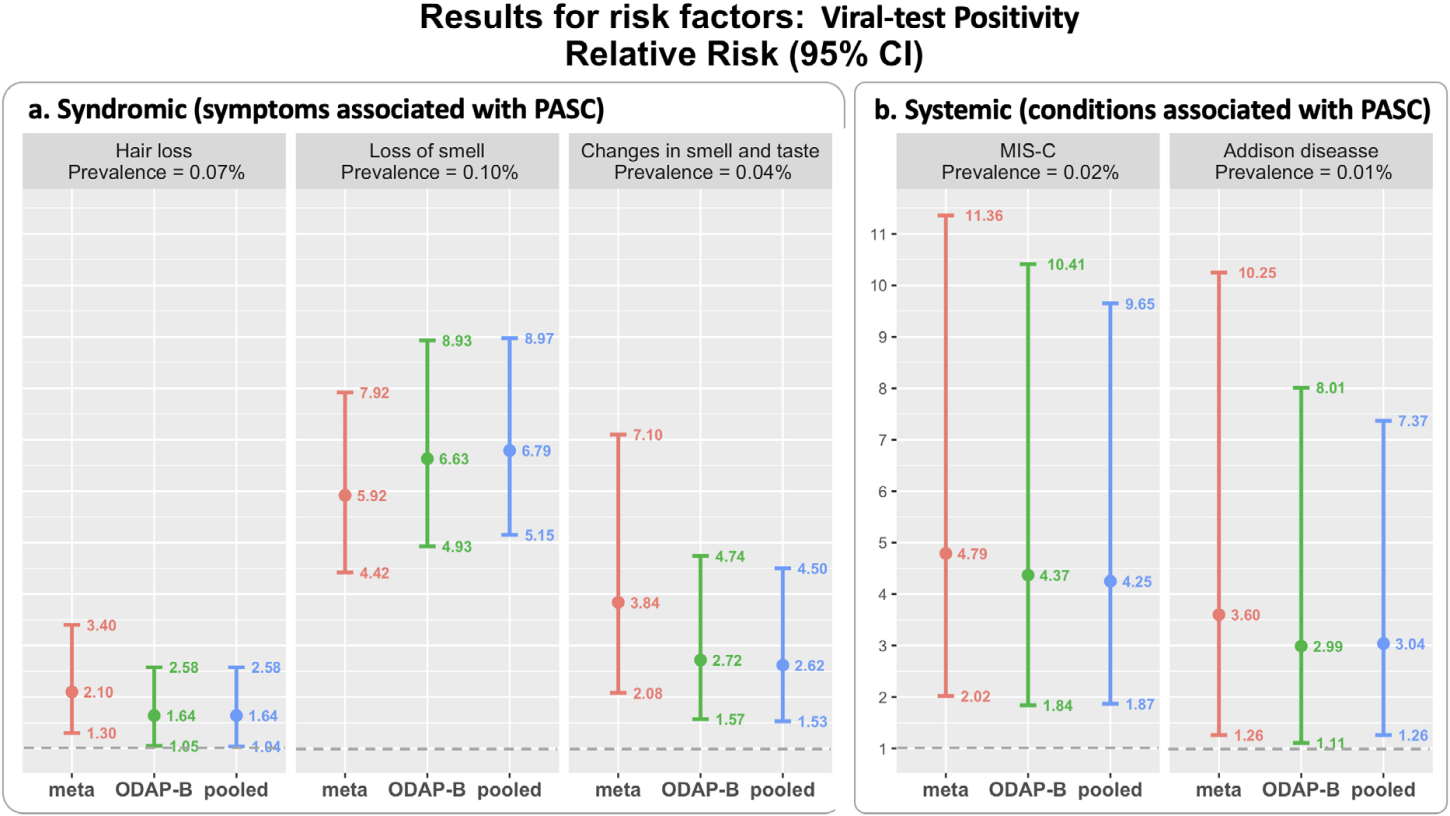
Comparison between the estimates of the risk factor, viral test positivity, with pooled method (blue), proposed ODAP‐B (green), and meta‐analysis method (red) using the real‐world data on postacute sequelae of SARS‐CoV‐2 infection (PASC) in 184 501 children across eight national clinical sites. The dashed horizontal line (gray) represents a relative risk value of one, indicating that there is no difference in the risk of experiencing PASC conditions between the two groups (positive vs. negative).

Detailed characteristics of the study sample are summarized in Table A1 in Section S2. The outcome is one of the five features (i.e., clinical outcomes/conditions) related to PASC identified by earlier investigations [34], including three syndromic (symptoms associated with PASC) features (changes in smell and taste, loss of smell, and hair loss) and two systemic (conditions associated with PASC) features (a multisystem inflammatory syndrome in children [MIS‐C] and Addison disease/adrenal insufficiency), all of which were previously shown to be clinical features of pediatric PASC [34]. The outcome assessment period spanned from 28 to 179 days after cohort entrance. The prevalence values of these outcomes are presented in Figure 4.

The exposure is positive COVID‐19 viral test and the confounding variables to be adjusted in the regression model include age at cohort entrance, sex (male vs. female), race/ethnicity (Hispanic, Non‐Hispanic White, Non‐Hispanic Black/African‐American, Non‐Hispanic Asian/Pacific Islander, Other/Unknown, or Multiple), COVID‐19 testing location (emergency department, inpatient, outpatient clinic, or outpatient testing facility), diagnosis date of the outcome (i.e., PASC conditions), and Pediatric Medical Complexity Algorithm (PMCA). PMCA is a methodology developed to identify and stratify children by level of medical complexity. We used the PMCA Version 2.0 [35] to categorize children as having no chronic condition (PMCA = 0), noncomplex chronic condition (PMCA = 1), or complex chronic condition comorbidities (PMCA = 2). We considered diagnoses up to 3 years before cohort entrance. Children were assigned to the complex chronic condition category if they had conditions that affected > 2 body systems (e.g., endocrine and renal), a progressive chronic condition (e.g., cystic fibrosis), or evidence of malignancy [35, 36].

For each of the five outcomes, we conducted the analysis to estimate the RR and 95% confidence interval (CI) of the risk factor, viral positivity (yes vs. no). The results are shown in Figure 4. We compared three methods, including the pooled method (top, blue), the proposed ODAP‐B method (middle, green), and the meta‐analysis method (bottom, red). *Panel a* on the left contains the results of the three syndromic features and the *panel b* on the right presents the results of two systemic PASC features. Each panel lists the results of the estimated RR of the features. In each forest plot, we can observe that the ODAP‐B method provides a closer estimate of the RR of PCR positivity compared with the one obtained by the meta‐analysis method. For most of the analyses, excluding loss of smell, the ODAP‐B method is more efficient (narrower CI) than the meta‐analysis method.

For the PASC syndromic conditions, the estimated RR of viral test positivity for having hair loss is 1.64 (95% CI: [1.05, 2.58]). This means that children with a positive PCR or antigen test are 64% more likely to have hair loss compared to children with a negative test. The estimated RR of viral test positivity for experiencing loss of smell is 6.63 (95% CI: [4.93, 8.93]), which means that children with a positive PCR or antigen test are over 6 times more likely to experience loss of smell compared to children with a negative test. The estimated RR of viral test positivity for experiencing changes in smell and taste is 2.72 (95% CI: [1.57, 4.74]), which means that children with a positive PCR or antigen test are about three times more likely to experience changes in smell and taste compared to children with a negative test.

For the PASC systemic conditions, the estimated RR of viral test positivity for having MIS‐C is 4.37 (95% CI: [1.84, 10.41]), which means that children with a positive PCR or antigen test are over 4 times more likely to be diagnosed with MIS‐C compared to children with a negative test. The estimated RR of viral test positivity for having Addison disease is 2.99 (95% CI: [1.11, 8.01]), which means that children with a positive PCR or antigen test are nearly three times more likely to be diagnosed with Addison disease compared to children with a negative test.

By comparing with a number of publications studying PASC in children, the findings of applying our methods, such as the direction and the statistical significance of the test positivity effects on the PASC features are consistent with the published studies [28, 37].

### 4.2 Use Case 2: Application With Decentralized Datasets of 452 160 Children From 13 Sites Within PEDSnet, Optum's Data From Janssen, and OneFlorida+ Clinical Research Consortium

To demonstrate the feasibility of using distributed methods in a decentralized network, we also applied our ODAP‐B method using data from five additional databases: Optum de‐identified EHR dataset from Janssen, Optum's de‐identified Clinformatics Data Mart Database (database of administrative health claims) from Janssen, and three databases from the OneFlorida+ Clinical Research Consortium. Patient‐level data from these databases are not available to us, resulting in a decentralized setting where only aggregated data can be shared. Using the *same* study design (i.e., cohort definition, inclusion criteria, covariates, outcomes), we analyzed a total of 452 160 children from all 13 sites (eight from PEDSnet, two from Janssen's databases, and three from OneFlorida+) for four of the five outcomes in use case 1: hair loss, loss of smell, change in smell and taste, and Addison disease. There were not enough cases of MIS‐C in the OneFlorida+ data, so the cohort sample size for MIS‐C as an outcome was 430 565.

**FIGURE 5:**
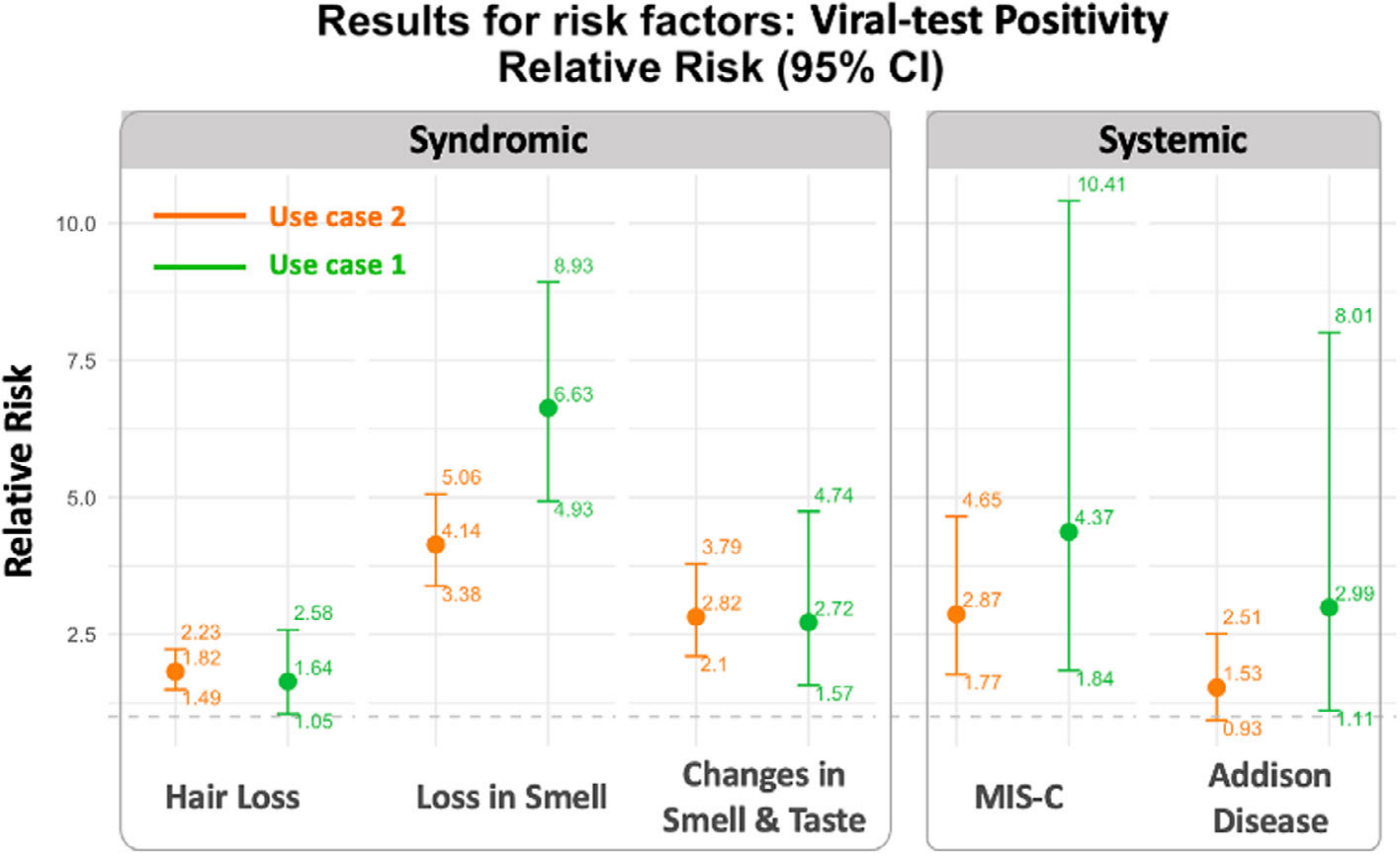
Estimates of the effect of the risk factor, viral test positivity, with the proposed ODAP‐B algorithm using the real‐world data from 13 sites on postacute sequelae of SARS‐CoV‐2 infection (PASC) of 452 160 children (use case 2), compared with results from use case 1 (orange: Use case 2; green: Use case 1 for comparison).

Figure 5 summarizes the estimated effect of viral test positivity and the corresponding 95% CI. The results of this decentralized use case indicate that viral test positivity is significantly associated with hair loss, loss of smell, and change in smell and taste, after controlling for variables such as age, gender, PMCA, and study entrance time. These findings are consistent with those from use case 1 (shown in green in Figure 5), and the confidence intervals are narrower than in the first use case due to the inclusion of more children from the Optum data and OneFlorida+ databases in the analysis. No significant association was found between viral test positivity and Addison disease.

## 5 Discussion

In this paper, we propose the One‐shot distributed modified algorithm for Poisson regression with binary data (ODAP‐B) method for the analysis of rare binary outcomes, providing an estimated RR with efficient sandwich variance estimates. Our method requires only one round of communication as the meta‐analysis method for the data contributors. In the simulation studies, the proposed method was shown to have higher accuracy than the traditional two‐step meta‐analysis when the outcome is relatively rare, which has a smaller relative bias with respect to the pooled method, suggesting its greater utility in rare‐event contexts. In addition, we applied the proposed ODAP‐B method to real‐world data analysis of PEDSnet, Optum from Janssen, and OneFlorida+ to study the effect of COVID‐19 viral test positivity on PASC features.

The proposed method has some limitations that offer opportunities for future development and evaluation. First, our algorithm utilizes the concept of constructing a surrogate likelihood function to approximate the pooled data likelihood. The pooled likelihood implicitly assumes that the associations between covariates and the outcome are homogeneous across hospitals, which can be evaluated or quantified using the Cochran's *Q* test [38] or the $I^{2}$ value [39]. However, given the complexity of real‐world data, the associations could be heterogeneous due to various factors, such as geographical variability, variations in patients' characteristics, and regional differences in practice patterns. Some efforts have been made to account for the potential heterogeneous data distributions across sites [40, 41, 42]. In the future, we plan to extend the algorithm to incorporate site‐specific effects and covariates to account for the heterogeneity between sites. Second, in addition to the between‐site heterogeneity, intrasite heterogeneity (subpopulations within a site) is a practical and important problem that needs to be considered. Third, to remove the potential first‐order bias in the MLE estimators of the effects of interest, we plan to adopt Firth's correction approach on the modified Poisson regression [43].

In future work, we plan to extend our method to handle high‐dimensional regression. This will involve incorporating regularization techniques such as LASSO or ridge regression to manage the increased complexity and prevent overfitting. Additionally, we aim to develop efficient algorithms that can scale with the number of features while maintaining computational feasibility. By addressing these challenges, we hope to enhance the applicability of our method to a broader range of datasets and improve its performance in high‐dimensional settings.

We believe that ODAP‐B is a significant contribution to the new generation of distributed research networks. As a powerful tool in modeling the risk factors of binary outcomes, the ODAP‐B method facilitates a collaborative environment by providing accurate estimation, privacy‐preserving features, and efficient communication.

## Author Contributions

J.T. and Y.C. designed methods and experiments. J.R., J.B., R.J., K.J.K., K.M.P., and C.B.F. provided the datasets for data analysis. Y.C. guided the theoretical development and dataset generation for the simulation study. L.L. and J.T. generated the simulation datasets, and conducted simulation experiments. J.T., J.R., and X.L. conducted data analysis. All authors interpreted the results and provided instructive comments. J.T. and L.L. drafted the main manuscript. All coauthors provided critical edits to the early draft and approved the final version of the manuscript.

## Conflicts of Interest

Dr. Jhaveri is a consultant for AstraZeneca, Seqirus, and Dynavax, and receives an editorial stipend from Elsevier. Other authors have no competing interests to declare.

## Supporting information


**Data S1** Supporting Information.

**Data S2** Supporting Information.

**Data S3** Supporting Information.

## Data Availability

Data for this study are unavailable due to patient privacy concerns. The aggregated data that support the findings of this study are available from the corresponding author upon request.
