# Supplementary material for: Advancing Interpretable Regression Analysis for Binary Data: A Novel Distributed Algorithm Approach
Source: Stat Med. 2024 Nov 3;43(29):5573–82. doi: 10.1002/sim.10250 (PMC11588971; doi:10.1002/sim.10250)
Supplement: Supplementary file 1 — Data S1 Supporting Information. [file SIM-43-5573-s003.docx]

**Supplementary materials for “Advancing Interpretable Regression Analysis for Binary Data: A Novel Distributed Algorithm Approach”**

Table of Contents

[Section 1. Map of the national data sources for use cases 1 and 2. 2](#_Toc172561519)

[Section 2. Summary characteristics of the PASC patients from 8 sites. 3](#_Toc172561520)

[Section 3. Additional Simulation Study 6](#_Toc172561521)

### Section 1. Map of the national data sources for use cases 1 and 2.

**
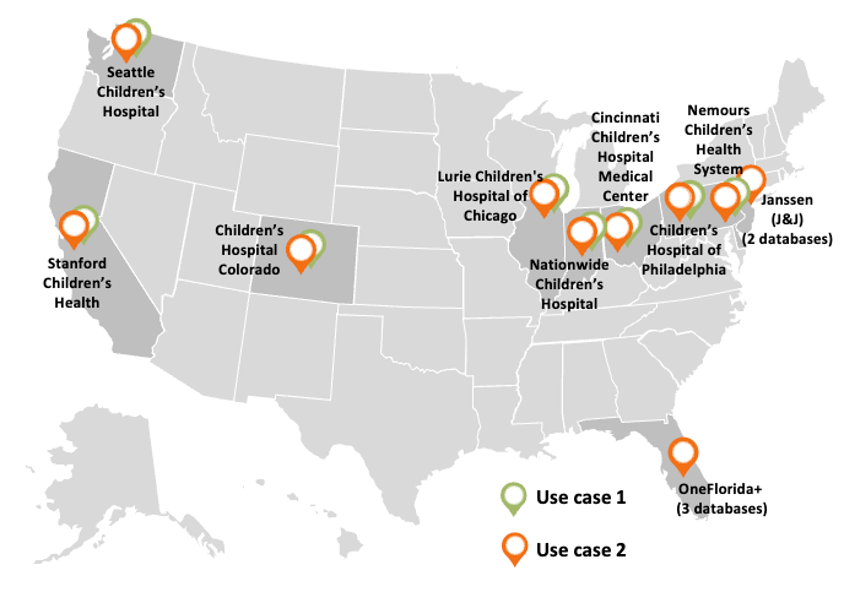
**

- Use case 1: **centralized** data from PEDSnet
  - Participating sites: nine pediatric hospitals from the PEDSnet (including Cincinnati Children's Hospital Medical Center, Seattle Children's Hospital, Stanford Children’s Health, Children’s Hospital Colorado, Lurie Children's Hospital of Chicago, Nemours Children’s Health System, Nationwide Children’s Hospital, Children’s Hospital of Philadelphia) contributed centralized data, hosted at the Children's Hospital of Philadelphia (CHOP)
- Use case 2: **decentralized** data from PEDSnet, Janssen, and OneFlorida+.
  - Participating sites: Janssen Pharmaceutical Companies of Johnson & Johnson and the OneFlorida+ Clinical Research Network (i.e., another network like PEDSnet with 14 different partners across Florida, Georgia, and Alabama

### Section 2. Summary characteristics of the PASC patients from 8 sites.

| Characteristic | |  | PCR Test Results | |  |
| --- | --- | --- | --- | --- | --- |
|  |  | **Overall**  **(n=184,501)** | **Negative**  **(n=168,107)** | **Positive**  **(n=16,394)** | **Standardized Difference** |
| Age at cohort entrance, years, n (%) | <1 | 16,848 (9.1%) | 15,630 (9.3%) | 1,218 (7.4%) | 0.24 |
|  | 1-4 | 53,936 (29.2%) | 50,322 (29.9%) | 3,614 (22.0%) |  |
|  | 5-11 | 29,715 (16.1%) | 26,512 (15.8%) | 3,203 (19.5%) |  |
|  | 12-15 | 24,013 (13.0%) | 21,024 (12.5%) | 2,989 (18.2%) |  |
|  | 16-20 | 59,989 (32.5%) | 54,619 (32.5%) | 5,370 (32.8%) |  |
| Sex, n (%) | Female | 87,237 (47.3%) | 79,206 (47.1%) | 8,031 (49.0%) | 0.04 |
| Race/ethnicity, n (%) | Hispanic | 29,186 (15.8%) | 25,983 (15.5%) | 3,203 (19.5%) | 0.19 |
|  | Non-Hispanic White | 95,721 (51.9%) | 88,051 (52.4%) | 7,670 (46.8%) |  |
|  | Non-Hispanic Black/African-American | 29,927 (16.2%) | 26,585 (15.8%) | 3,342 (20.4%) |  |
|  | Non-Hispanic Asian/Pacific Islander | 8,048 (4.4%) | 7,572 (4.5%) | 476 (2.9%) |  |
|  | Other/  Unknown | 14,117 (7.7%) | 13,031 (7.8%) | 1,086 (6.6%) |  |
|  | Multiple | 7,502 (4.1%) | 6,885 (4.1%) | 617 (3.8%) |  |
| Institution, n (%) | Site A | 34,641 (18.8%) | 30,658 (18.2%) | 3,983 (24.3%) | 0.28 |
|  | Site B | 37,852 (20.5%) | 34,422 (20.5%) | 3,430 (20.9%) |  |
|  | Site C | 26,749 (14.5%) | 24,242 (14.4%) | 2,507 (15.3%) |  |
|  | Site D | 11,334 (6.1%) | 10,731 (6.4%) | 603 (3.7%) |  |
|  | Site E | 30,615 (16.6%) | 27,788 (16.5%) | 2,827 (17.2%) |  |
|  | Site F | 23,470 (12.7%) | 21,405 (12.7%) | 2,065 (12.6%) |  |
|  | Site G | 9,422 (5.1%) | 9,159 (5.4%) | 263 (1.6%) |  |
|  | Site H | 10,418 (5.6%) | 9,702 (5.8%) | 716 (4.4%) |  |
| PMCA (Chronic conditions), n (%) | None (0) | 125,226 (67.9%) | 113,810 (67.7%) | 11,416 (69.6%) | 0.05 |
|  | Non-complex (1) | 29,734 (16.1%) | 27,173 (16.2%) | 2,561 (15.6%) |  |
|  | Complex (2) | 29,541 (16.0%) | 27,124 (16.1%) | 2,417 (14.7%) |  |
| Time period of cohort entrance, n (%) | Mar 2020-Jun 2020 | 12,408 (6.7%) | 11,956 (7.1%) | 452 (2.8%) | 0.44 |
|  | Jul 2020-Oct 2020 | 36,816 (20.0%) | 35,267 (21.0%) | 1,549 (9.4%) |  |
|  | Nov 2020-Feb 2021 | 42,750 (23.2%) | 37,255 (22.2%) | 5,495 (33.5%) |  |
|  | Mar 2021-Jun 2021 | 37,971 (20.6%) | 34,834 (20.7%) | 3,137 (19.1%) |  |
|  | Jul 2021-Oct 2021 | 48,848 (26.5%) | 43,746 (26.0%) | 5,102 (31.1%) |  |
|  | Nov 2021- Dec 31^st^ 2021 | 5,708 (3.1%) | 5,049 (3.0%) | 659 (4.0%) |  |
| Test Location, n (%) | Emergency Department | 36,082 (19.6%) | 32,840 (19.5%) | 3,242 (19.8%) | 0.17 |
|  | Inpatient | 12,993 (7.0%) | 12,369 (7.4%) | 624 (3.8%) |  |
|  | Outpatient Clinic | 45,117 (24.5%) | 41,357 (24.6%) | 3,760 (22.9%) |  |
|  | Outpatient Testing Facility | 90,309 (48.9%) | 81,541 (48.5%) | 8,768 (53.5%) |  |

Table A1 presents the summary characteristics of the PASC patients from 8 sites. In the model, the exposure of interest is positive COVID-19 viral test and the variables to be adjusted in the regression model include age at cohort entrance, sex, race/ethnicity, testing location (emergency department, inpatient, outpatient clinic, or outpatient testing facility), diagnosis date of the outcome, and Pediatric Medical Complexity Algorithm (PMCA)^1^.

The total number of patients is 184,501, with 168,107 (91.1%) in negative group and 16,394 (8.9%) in positive group. The standardized differences of the variables are presented in the last column of Table 2, ranging from 0.04 to 0.44. These values indicate that the baseline characteristics between the two groups (i.e., test positive group vs. test negative group) are relatively balanced.

1 Simon TD, Haaland W, Hawley K, Lambka K, Mangione-Smith R. Development and validation of the Pediatric Medical Complexity Algorithm (PMCA) version 3.0. *Acad Pediatr* 2018; **18**: 577–80.

### Section 3. Additional Simulation Study

We further examined the preservation of type-I error of proposed method. Specifically, we set β = 0 to examine the preservation of type-I error rates, by keeping all other parameters the same. The following table presents the summary of the results, which has also been added to the revised Supplementary Materials Appendix Section 3.

We found that for most of the settings, the proposed method maintains an acceptable level of type I error and yields values closer to those of the pooled analysis compared to the meta-analysis method.

| K | $\boldsymbol{\alpha}$ | $\boldsymbol{\beta}$ | Pooled analysis | Meta-analysis | Proposed  ODAP-B |
| --- | --- | --- | --- | --- | --- |
| 5 | $\alpha$= -2 | $\boldsymbol{\beta}$ == 0 | 0.015 | 0.024 | **0.023** |
| 50 |  |  | 0.009 | 0.047 | **0.021** |
| 5 | $\alpha$ = -5 |  | 0.030 | 0.118 | **0.048** |
| 50 |  |  | 0.022 | 0.251 | **0.111** |

*Table A1. The estimated type-I errors under various settings of simulation studies of pooled analysis, meta-analysis, and the proposed ODAP-B method.*
